# Risk factors of hospitalization for any medical condition among patients with prior emergency department visits for mental health conditions

**DOI:** 10.1186/s12888-020-02835-2

**Published:** 2020-09-03

**Authors:** Louise Penzenstadler, Lia Gentil, Guy Grenier, Yasser Khazaal, Marie-Josée Fleury

**Affiliations:** 1 Douglas Hospital Research Center, Douglas Mental Health University Institute, McGill University, 6875 LaSalle Boulevard, Montréal, Québec, H4H 1R3 Canada; 2 Hôpitaux Universitaires Genève, Département de psychiatrie, Service d’addictologie, Rue du Grand-Pré 70c, 1202 Geneva, Switzerland; 3 Institut universitaire sur les dépendances du Centre intégré universitaire de santé et des services sociaux du Centre-Sud-de-l’Île-de-Montréal, 950 Louvain East, Montréal, Québec, H2M 2E8 Canada; 4 Centre hospitalier universitaire vaudois, Département de psychiatrie, Service de médecine des addictions, Policlinique d’addictologie, Rue du Bugnon 23, 1011 Lausanne, Switzerland; 5 Département de psychiatrie et d’addictologie, Université de Montréal, 2900 bld Eduard-Montpetit, Montréal, Québec, H3T1J4 Canada

**Keywords:** Hospitalization; mental disorders; substance related disorders; risk factors, Needs factors, Predisposing factors, Enabling factors, Medical conditions, Predictors, Service use

## Abstract

**Background:**

This longitudinal study identified risk factors for frequency of hospitalization among patients with any medical condition who had previously visited one of six Quebec (Canada) emergency departments (ED) at least once for mental health (MH) conditions as the primary diagnosis.

**Methods:**

Records of *n* = 11,367 patients were investigated using administrative databanks (2012–13/2014–15). Hospitalization rates in the 12 months after a first ED visit in 2014–15 were categorized as no hospitalizations (0 times), moderate hospitalizations (1–2 times), and frequent hospitalizations (3+ times). Based on the Andersen Behavioral Model, data on risk factors were gathered for the 2 years prior to the first visit in 2014–15, and were identified as predisposing, enabling or needs factors. They were tested using a hierarchical multinomial logistic regression according to the three groups of hospitalization rate.

**Results:**

Enabling factors accounted for the largest percentage of total variance explained in the study model, followed by needs and predisposing factors. Co-occurring mental disorders (MD)/substance-related disorders (SRD), alcohol-related disorders, depressive disorders, frequency of consultations with outpatient psychiatrists, prior ED visits for any medical condition and number of physicians consulted in specialized care, were risk factors for both moderate and frequent hospitalizations. Schizophrenia spectrum and other psychotic disorders, bipolar disorders, and age (except 12–17 years) were risk factors for moderate hospitalizations, while higher numbers (4+) of overall interventions in local community health service centers were a risk factor for frequent hospitalizations only. Patients with personality disorders, drug-related disorders, suicidal behaviors, and those who visited a psychiatric ED integrated with a general ED in a separate site, or who visited a general ED without psychiatric services were also less likely to be hospitalized. Less urgent and non-urgent illness acuity prevented moderate hospitalizations only.

**Conclusions:**

Patients with severe and complex health conditions, and higher numbers of both prior outpatient psychiatrist consultations and ED visits for medical conditions had more moderate and frequent hospitalizations as compared with non-hospitalized patients. Patients at risk for frequent hospitalizations were more vulnerable overall and had important biopsychosocial problems. Improved primary care and integrated outpatient services may prevent post-ED hospitalization.

## Introduction

Frequent use of inpatient treatment is a common occurrence among patients with mental disorders (MD) [1–3]. Most MD, including schizophrenia [4, 5] and mood disorders [6–8] as well as substance use disorders (SUD) [9, 10] involve a high risk of relapse which may explain frequent hospitalizations. Those with MD also experience high hospitalization rates for medical and surgical conditions [11] related to illnesses such as diabetes [12], heart problems and pneumonia [13, 14]. The frequent co-occurrence of MD with chronic physical illnesses [15–17] may affect access and challenge the capacity of primary care to provide adequate services [18], resulting in increased hospitalization rates [15–17].

Frequent hospitalizations account, in part, for the high costs of psychiatric treatment [19–21]. Understanding which risk factors predict frequency of patient hospitalizations among individuals with MD is important for improving care management and service organization. The Andersen Behavioral Model [22], one of the most widely used conceptual frameworks in studies of health service utilization, may serve to identify various risk factors for hospitalization. In this model, variables are categorized as needs, predisposing and enabling factors [22]. Needs factors include diagnoses and other clinical variables; predisposing factors refer to sociodemographic and economic status (e.g. age, sex), while enabling factors include variables with particular impact on healthcare use (e.g. access to care and continuous follow-up) [23].

Studies have examined risk factors for frequency of hospitalization in the general population [11, 24, 25]. The following needs factors were associated with high hospitalization rates: MD diagnoses [24], especially depressive symptoms [26] and personality disorders [27], co-occurring MD and physical illnesses [11], and co-occurring SUD and physical illnesses [25]. One study [24] found that older age (predisposing factors) and possession of health insurance (enabling factors) were linked to high hospitalization rates. However, other key factors which may influence hospitalization have been underexplored. These include enabling factors such as continuity of care, access to specialized ambulatory and primary care services and community treatment, and predisposing factors such as social deprivation. Moreover, no study to our knowledge has examined patient subgroups with MD or patients visiting ED for mental health (MH) conditions in terms of their frequency of hospitalization for medical conditions in a 12-month period. Examining risk factors of hospitalization for medical conditions in this patient cohort may capture the overall impact of this subpopulation on hospitalization rates, either for MH or physical health conditions. Reasons for inadequacy of care among these patients may be identified, allowing for further development of recommendations aimed at improving services.

Using the Andersen Model [22], this study assessed risk factors of hospitalization for any medical condition (no hospitalization (0 times), moderate hospitalizations (1–2 times), and frequent hospitalizations (3+ times)) in a 12 month-period after a first patient emergency department (ED) visit in 2014–15 for MH conditions as the primary diagnosis. Risk factors were gathered over a 2-year period prior to the first patient ED visit. Based on literature which identified key links between hospitalization rates and diagnoses, we hypothesized that frequent hospitalizations would be more strongly associated with needs factors, compared with enabling and predisposing factors.

## Methods

### Study population and design

This longitudinal study included 11,367 patients identified through clinical administrative databanks and followed over a three-year period (2012–13/2014–15). Participants were 12 years old or older and eligible for Quebec (Canada) healthcare insurance. They visited one of six Quebec ED at least once between April 1, 2014 and March 31, 2015 (index year) for MH conditions (including SUD and suicidal behaviors) as a primary diagnosis. The six selected ED operated in various local health networks, which represented three of the largest cities in Quebec. The Access to Information Commission of Quebec and the ethics committee of a MH university institute approved this study.

### Data sources

Data for the study were collected from the *Régie d’Assurance Maladie du Québec* (RAMQ), the Quebec healthcare insurance databanks which contain medical administrative information including billing files for medical services provided by physicians. These files cover most outpatient medical activities as only 6% of physician billing occurs outside of the public system [28]. The RAMQ also integrates demographic and socioeconomic information, including material and social deprivation indices [29]. Other data included the hospitalization/discharge databank (*Maintenance et exploitation de données pour l’étude de la clientèle hospitalière:* MED-ECHO) and the Quebec emergency databank (Banque de données commune des urgences: BDCU), adding pertinent complementary information (e.g., having a family physician or not, illness acuity, patient reasons for visiting ED as evaluated by triage nurses). Finally, the local community health service center databank (*Système d’information clinique et administrative des centres locaux de services communautaires*) provided additional data on biopsychosocial services offered by the Quebec public primary care system, including medical interventions provided by salaried general practitioners (GP) and primary care MH services.

### Variables

The dependent variable, frequency of patient hospitalization in 2014–15, included three categories: no hospitalization (0 times), moderate hospitalizations (1–2 times) and frequent hospitalizations (3+ times). While no consensus exists on the definition of frequent hospitalization, 3 hospitalizations per year has been the usual benchmark [3, 30–32]. Patients with 1–2 or moderate numbers of hospitalizations were considered at possible risk for frequent hospitalizations. The no-hospitalization group was used as a case control for the other group comparisons.

**Fig. 1:**
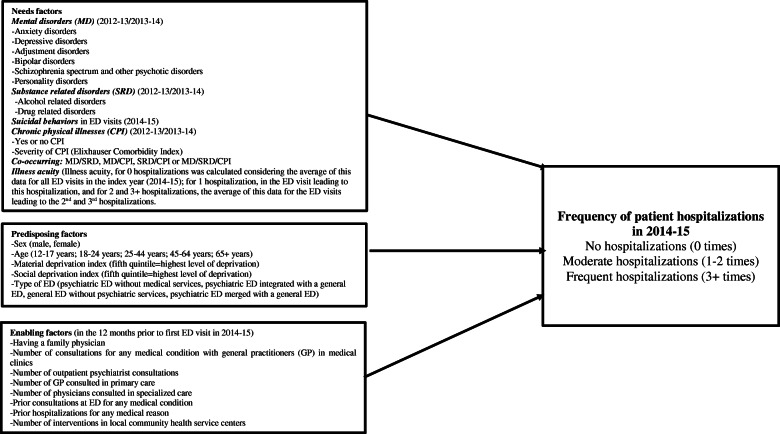
Conceptual framework of factors tested for associations with no, moderate, and frequent hospitalizations for any medical condition in 2014–15 (for patients who visited Quebec emergency departments (ED) at least once in 2014–15 for mental health (MH) reasons)

Based on the Andersen Behavioral Model (Fig. 1), needs factors consisted of various MD, including substance related disorders (SRD), based on the International Classification of Diseases Ninth Revision (ICD-9) from the RAMQ databank, or the Tenth Revision (ICD-10-CA) from the MED-ECHO and BDCU databanks. MD included: anxiety disorders, depressive disorders, adjustment disorders (common MD); and bipolar disorders, personality disorders, schizophrenia spectrum and other psychotic disorders (serious MD). SRD encompassed: alcohol related disorders (alcohol use disorders, alcohol induced disorders, alcohol intoxication) and drug related disorders (drug use disorders, drug induced disorders, drug intoxication). Suicidal behaviors, related to both suicidal ideation and attempt, as the reason for ED visit was extracted from the 2014–15 BDCU. Based on the Elixhauser Comorbidity Index [33], having chronic physical illnesses or not, and level of severity (0 to 3+), were recorded. Different combinations of co-occurring disorders involving MD, SRD and chronic physical illnesses were included. MD and SRD had to be recorded at least once in the two years (2012–13/2013–14) prior to the index year; and chronic physical illnesses twice yearly in the RAMQ databank, or once in the MED-ECHO, based on previous research [34]. As MD usually involve a course lasting more than one year, a two-year period was chosen as the standard for identifying patients diagnosed with MD prior to a first ED visit in 2014–15 [35–37]. Illness acuity at ED visit was also considered, based on the Canadian Triage Acuity Scale [38]. Illness acuity levels range from 1 (most urgent) to 5 (least urgent) and determine ED treatment order based on symptoms. For this study, they were grouped into levels 1–2 (immediate and very urgent), 3 (urgent) and 4–5 (less urgent and non-urgent). Ambulatory care is considered more appropriate than ED treatment for triage levels 4 and 5 [38]. For patients with no hospitalization following an ED visit, average illness acuity for all ED visits in 2014–15 was considered. Whereas for patients with 1–2 hospitalizations, or those with 3+ hospitalizations, illness acuity at the most recent ED visit prior to last hospitalization in each group was calculated.

Predisposing factors were sex (male, female), age (12–17 years, 18–24 years, 25–44 years, 45–64 years, and 65+ years) and material and social deprivation indices [29]. Deprivation indices combine six indicators from the 2011 Canadian census for the smallest geographical units from which census data are retrieved. The material deprivation index considers the employment-to-population ratio, the proportion of individuals without a high school diploma, and average income [29]. The social deprivation index includes proportions of individuals living alone, single-parent families, and individuals separated, divorced, or widowed [29]. Both indices are classified in quintiles, with the fifth quintile representing highest level of deprivation. The types of ED were classified in four groups: psychiatric ED without medical services within a MH university institute, psychiatric ED integrated with a general ED located in a separate site, general ED without psychiatric services, and psychiatric ED merged with a general ED.

Enabling factors included having a family physician (2014–15), and use of the following services in the 12 months preceding the first 2014–15 ED visit: number of consultations for any medical condition with a general practitioner (GP) in medical clinics (0, 1–3 and 4+ consultations); number of outpatient psychiatrist consultations (0, 1–3, 4–6, and 7+ consultations); number of GP consulted in primary care (0, 1–2, 3+); number of physicians consulted in specialized care (0, 1–2, 3+); prior consultations at ED for any medical condition: prior hospitalizations for any medical condition; and number of interventions in local community health service centers (0, 1–3 and 4+ interventions), i.e. primary care biopsychosocial services.

### Statistical analysis

The data were first imputed, showing less than 1% missing data which were replaced by the means [39, 40]. Descriptive analyses were performed including two-way frequency tables for each independent variable, in association with each of the three levels of the dependent variable (no hospitalizations, moderate hospitalizations, and frequent hospitalizations). Bivariate analyses were conducted, and independent variables found significant (*p* < .05) were included in the multivariable model. Collinearity statistics were also tested using variance inflation factors (VIF) and tolerance tests, with 5 as the maximum level of VIF. Only independent variables found without collinearity were entered in the model. A multivariable hierarchical multinomial logistic regression was run. Needs, predisposing and enabling factors were entered in the model in that order, based on previous studies concerning service use [22, 41–43]. AIC (Akaike’s Information Criterion) and BIC (Bayesian information criterion ) were used as criteria for the selection of the model. A stepwise forward method was also conducted for the estimation of parameters in the multinomial hierarchical logistic regression model. All analyses were performed using SPSS 24.0.

## Results

Tables 1, 2 and 3 present sample characteristics. In terms of needs factors for the two years prior to the first 2014–15 ED visit, 67% of study participants had been diagnosed with MD and 15% with SRD. Of those with MD, 40% had common MD (e.g. depressive disorders, anxiety disorders), and 26% serious MD (schizophrenia spectrum and other psychotic disorders, bipolar disorders). Forty percent of patients presented with suicidal behaviors in the ED visit during the index year 2014–15. Approximately one third (31%) were diagnosed with chronic physical illnesses, although severity levels were low in 77% of cases according to the Elixhauser comorbidity index (index 0). The most common co-occurring disorders were MD/chronic physical illnesses (34%) and MD/SRD (18%). Most patients (67%) presented at ED with illness acuity levels of 4 or 5 (less urgent and non-urgent). Regarding predisposing factors, 38% of patients were between 25 and 44 years of age, half of them female (51%). Material deprivation levels varied little among patients. However, 54% lived in the two most socially deprived areas (levels 4–5). Most patients (29%) had consulted a psychiatric ED without medical services. Concerning enabling factors, 49% of patients reported having a family physician (2014–15). In the 12 months prior to their first 2014–15 ED visit, 30 % had not consulted a GP in primary care; 31% had seen one or two physicians, and 39% three or more. Of the 46% who consulted outpatient psychiatrists, 25% made 7+ consultations. Forty-two percent had not consulted a physician in specialized care; 48% had been seen by one or two physicians, and 10% by three or more. Seventy-three percent made prior ED visits, and 22% had prior hospitalizations for any medical condition. The overall number of interventions in local community health service centers was low, with most patients (60%) receiving no intervention at all.

**Table 1:** Variables related to needs factors predicting frequency of patient hospitalizations for any medical condition in 2014–15

Characteristics	Total N (%)	No hospitalizations (0 times) N (%)	Moderate hospitalizations (1–2 times) N (%)	Frequent hospitalizations (3+ times) N (%)	p
**Overall**	11,367 (100)	6424 (56.5)	4192 (36.9)	751 (6.6)	
**Needs factors**					
Mental disorders (MD) (2012–2014)	7624 (67.1)	4176 (65.0) ^2^	2931 (69.9) ^1^	517 (68.8)	.00
Common MD	4569 (40.2)	2484 (38.7) ^2^	1783 (42.5) ^1^	302 (40.2)	.00
Depressive disorders	1468 (12.9)	722 (11.2) ^2,3^	636 (15.2) ^1^	110 (14.6) ^1^	.00
Anxiety disorders	3870 (34.0)	2155 (33.5)	1462 (34.9)	253 (33.7)	.36
Adjustment disorders	3841 (33.8)	2183 (34.0)	1415 (33.8)	243 (32.3)	.67
Serious MD	3004 (26.4)	1480 (23.0) ^2,3^	1293 (30.8) ^1^	231 (30.8) ^1^	.00
Schizophrenia spectrum and other psychotic disorders	2144 (18.9)	1074 (16.7) ^2,3^	905 (21.6) ^1^	165 (22.0) ^1^	.00
Bipolar disorders	1564 (13.8)	764 (11.9) ^2,3^	683 (16.3) ^1^	117 (15.6) ^1^	.00
Personality disorders	1442 (12.7)	925 (14.4) ^2,3^	453 (10.8) ^1^	64 (8.5) ^1^	.00
Substance-related disorders (SRD) (drug and alcohol) (2012–2014)	1805 (15.3)	1036 (15.2)	657 (15.5)	112 (14.9)	.86
Alcohol related disorders	1112 (9.8)	562 (8.7) ^2^	468 (11.2) ^1^	82 (10.9)	.00
Drug related disorders	1086 (9.6)	678 (10.6) ^2,3^	357 (8.5) ^1^	51 (6.8) ^1^	.00
Suicidal behaviors in ED visits (2014–2015)	4512 (39.7)	2810 (43.7) ^2,3^	1456 (34.7) ^1^	246 (32.8) ^1^	.00
Chronic physical illnesses	3547 (31.2)	1471 (22.9) ^2,3^	1609 (38.4) ^1,3^	467 (62.2) ^1,2^	.00
Elixhauser Comorbidity Index (2012–2014) ^a^					.00
0	8713 (76.7)	5293 (81.6) ^2,3^	3049 (72.7) ^1,3^	425 (56.6) ^1,2^	.00
1	1120 (9.9)	617 (9.6)	414 (9.9)	89 (11.9)	.00
2	635 (5.6)	278 (4.3) ^2,3^	291 (6.9) ^1^	66 (8.8) ^1^	.00
3+	899 (7.9)	290 (4.5) ^2,3^	438 (10.4) ^1,3^	171 (22.8) ^1,2^	.00
Co-occurring MD/SRD	2084 (18.3)	726 (11.3) ^2,3^	1054 (25.1) ^1,3^	304 (40.5) ^1,2^	.00
Co-occurring MD/chronic physical illnesses	3843 (33.8)	1219 (19.0) ^2,3^	2073 (49.5) ^1,3^	551 (73.4) ^1,2^	.00
Co-occurring SRD/chronic physical illnesses	1047 (9.2)	260 (4.0) ^2,3^	571 (13.6) ^1,3^	216 (28.8) ^1,2^	.00
Co-occurring MD/SRD/chronic physical illnesses	903 (7.9)	175 (2.7) ^2,3^	522 (12.5) ^1,3^	206 (27.4) ^1,2^	.00
Illness acuity ^b^					.00
Levels 1 and 2 (immediate and very urgent)	1173 (10.3)	639 (9.9) ^2,3^	482 (11.5) ^1,3^	52 (6.9) ^1,2^	
Level 3 (urgent)	2556 (22.5)	1456 (22.7) ^3^	1003 (23.9) ^3^	94 (12.5) ^1,2^	
Levels 4 and 5 (less urgent and non-urgent)	7638 (67.3)	4326 (67.3) ^2,3^	2707 (64.6) ^1,3^	605 (80.6) ^1,2^	

χ^2^ Comparisons are produced for each row reporting percentages for categorical variables. Superscript indicates a significant difference at *p* < .05. For example, the percentage of individuals with MD having no hospitalizations was significantly different than the one having 1–2 hospitalizations. No hospitalizations = column 1, moderate hospitalizations [1, 2]=column 2, and frequent hospitalizations (3+ times) = column 3

^a^Chronic physical illnesses included: chronic pulmonary disease, cardiac arrhythmias, tumor and/or metastasis, renal disease, fluid electrolyte disorders, myocardial infarction, congestive heart failure, metastatic cancer, dementia, stroke, neurological disorders, liver disease, pulmonary circulation disorders, coagulopathy, weight loss, paralysis, AIDS/HIV

^b^Patients in the study sample had visited ED at least one time in 2014–15 for mental health conditions. For 0 hospitalizations, illness acuity was calculated considering the average of this data for all ED visits in the index year (2014–15); for 1 hospitalization, in the ED visit leading to this hospitalization; and for 2 and 3+ hospitalizations, the average of these data for the ED visits leading to the 2nd and 3rd hospitalizations

**Table 2:** Variables related to predisposing factors predicting frequency of patient hospitalizations for any medical condition in 2014–15

Characteristics	Total N (%)	No hospitalizations (0 times) N (%)	Moderate hospitalizations (1–2 times) N (%)	Frequent hospitalizations (3+ times) N (%)	p
**Overall**	11,367 (100)	6424 (56.5)	4192 (36.9)	751 (6.6)	
**Predisposing factors (2014–2015)**					
Age					.00
12–17 years	739 (6.5)	492 (7.7) ^2,3^	190 (4.5) ^1,3^	57 (7.6) ^1,2^	
18–24 years	1953 (17.2)	1067 (16.6)	751 (17.9)	135 (18.0)	
25–44 years	4330 (38.1)	2472 (38.5)	1569 (37.4)	289 (38.5)	
45–64 years	3128 (27.5)	1748 (27.2)	1185 (28.3)	195 (26.0)	
65 years+	1217 (10.7)	645 (10.0) ^2^	497 (11.9) ^1^	75 (10.0)	
Sex					.92
Male	5537 (48.7)	3128 (48.7)	2038 (48.6)	371 (49.4)	
Female	5830 (51.3)	3296 (51.3)	2154 (51.4)	380 (50.6)	
Deprivation Index:					
Material					.27
1: Least deprived	2402 (21.1)	1382 (21.5)	860 (20.5)	160 (21.3)	
2	1747 (15.4)	985 (15.3)	647 (15.4)	115 (15.3)	
3	2038 (17.9)	1153 (17.9)	761 (18.2)	124 (16.5)	
4	1978 (17.4)	1134 (17.7)	721 (17.2)	123 (16.4)	
5: Most deprived	2187 (19.2)	1213 (18.9)	834 (19.9)	140 (18.6)	
Not assigned ^a^	1015 (8.9)	557 (8.7)	369 (8.8)	89 (11.9)	
Social					.02
1: Least deprived	1367 (12.0)	771 (12.0)	502 (12.0)	94 (12.5)	
2	1257 (11.1)	719 (11.2)	456 (10.9)	82 (10.9)	
3	1540 (13.5)	907 (14.1) ^2^	532 (12.5) ^1^	110 (14.5)	
4	2528 (22.2)	1445 (22.5)	924 (22.0)	159 (21.2)	
5: Most deprived	3660 (32.2)	2025 (31.5) ^2^	1418 (33.8) ^1,3^	217 (28.9) ^2^	
Not assigned ^a^	1015 (8.9)	557 (8.7) ^3^	369 (8.8) ^3^	89 (11.9) ^1,2^	
Type of emergency department (ED)					.00
Psychiatric ED without medical services	3296 (29.0)	1829 (28.5)	1250 (29.8)	217 (28.9)	
Psychiatric ED integrated with a general ED located in a separate site	2039 (17.9)	1238 (19.3) ^2,3^	696 (16.6) ^1^	105 (14.0) ^1^	
General ED without psychiatric services	2871 (25.3)	1663 (25.9)	1041 (24.8)	167 (22.2)	
Psychiatric ED merged with a general ED	3161 (27.8)	1694 (26.4) ^2,3^	1205 (28.7) ^1,3^	262 (34.9) ^1,2^	

χ^2^ Comparisons were produced for each row reporting percentages for categorical variables. Superscript indicates a significant difference at p < .05. For example, the percentage of individuals who were 12–17 years old having no hospitalizations was significantly different than the group with 1–2 or 3+ hospitalizations. No hospitalizations (0 times) = column 1, moderate hospitalizations (1–2 times) = column 2, and frequent hospitalizations (3+ times) = column 3

^a^This corresponds to missing address or to residence in an area where index assignment is not available. An index cannot usually be assigned to residents of long-term health care units or homeless individuals

**Table 3:** Variables related to enabling factors predicting frequency of patient hospitalizations for any medical condition in 2014–15

Characteristics	Total N (%)	No hospitalization (0 times) N (%)	Moderate hospitalizations (1–2 times) N (%)	Frequent hospitalizations (3+ times) N (%)	p
**Overall**	11,367 (100)	6424 (56.5)	4192 (36.9)	751 (6.6)	
**Enabling factors**					
Having a family physician (2014–15)	5534 (48.7)	3157 (49.1)	2016 (48.1)	361 (48.1)	.54
Number of consultations for any medical condition with a general practitioner (GP) in private clinics ^a^					.00
0 visits	3536 (31.1)	1779 (27.7) ^2,3^	1461 (34.9) ^1,3^	296 (39.4) ^1,2^	
1–3 visits	4336 (38.1)	2565 (39.9) ^2,3^	1525 (36.4) ^1^	246 (32.8) ^1^	
4+ visits	3495 (30.7)	2080 (32.4) ^2,3^	1206 (28.8) ^1^	209 (27.8) ^1^	
Number of outpatient psychiatrist consultations ^a^					.00
0 consultations	6097 (53.6)	4653 (72.4) ^2,3^	1297 (30.9) ^1,3^	147 (19.6) ^1,2^	
1–3 consultations	1744 (15.3)	1010 (15.7) ^3^	664 (15.8) ^3^	70 (9.3) ^1,2^	
4–6 consultations	742 (6.5)	294 (4.6) ^2,3^	387 (9.2) ^1^	61 (8.1) ^1^	
7+ consultations	2784 (24.5)	467 (7.3) ^2,3^	1844 (44.0) ^1,3^	473 (63.0) ^1,2^	
Number of GP consulted in primary care ^a^					.00
0 GPs	3421 (30.1)	1866 (29.0) ^2^	1311 (31.3) ^1^	244 (32.5)	
1–2 GPs	3525 (31.0)	1897 (29.5) ^2^	1381 (32.9) ^1^	247 (32.9)	
3+ GPs	4421 (38.9)	2661 (41.4) ^2,3^	1500 (35.8) ^1^	260 (34.6) ^1^	
Number of physicians consulted in specialized care ^a^					.00
0 physicians	4726 (41.6)	3833 (59.7) ^2,3^	791 (18.9) ^1,3^	102 (13.6) ^1,2^	
1–2 physicians	5466 (48.1)	2176 (33.9) ^2,3^	2772 (66.1) ^1^	518 (69.0) ^1^	
3+ physicians	1175 (10.3)	415 (6.5) ^2,3^	629 (15.0) ^1^	131 (17.4) ^1^	
Prior consultations at emergency department (ED) for any medical condition ^a^	8256 (72.6)	4088 (36.0) ^2,3^	3448 (30.3) ^1,3^	720 (6.3) ^1,2^	.00
Prior hospitalizations for any medical condition ^a^	2461 (21.7)	1365 (21.2)	928 (22.1)	168 (22.1)	.49
Number of overall interventions in local community health service centers ^a^					
0 interventions	6806 (59.9)	4074 (63.4) ^2,3^	2387 (56.9) ^1,3^	345 (45.9) ^1,2^	
1–3 interventions	1749 (15.4)	980 (15.3)	668 (15.9)	101 (13.4)	
4+ interventions	2812 (24.7)	1370 (21.3) ^2,3^	1137 (27.1) ^1,3^	305 (40.6) ^1,2^	

χ^2^ Comparisons were produced for each row reporting percentages for categorical variables. Superscript indicates a significant difference at p < .05. For example, the percentage of individuals with 0 visits to the GP was significantly different than the one with 1–3 or 4+ visits. No hospitalizations (0 times) = column 1, moderate hospitalizations (1–2 times) = column 2, and frequent hospitalizations (3+ times) = column 3

^a^Measured in the 12 months prior to first ED visit in the index year (2014–2015)

Of the 11,367 ED users with MD, most were not hospitalized (56%), while 37% had 1 or 2 (27 and 10% respectively: moderate) hospitalizations, and 7% had 3+ (frequent) hospitalizations. Concerning the principal conditions for hospitalization, 56% were admitted for physical illnesses and 44% for MD, including 9% for SRD. The mean duration of hospital stay per admission was 28.5 days (SD = ±46.1) for those with moderate hospitalizations, and 72.0 days (SD = ±67.5) for those with frequent hospitalizations, who also had 4 hospitalizations on average, ranging from 1 to 28. Among patients hospitalized 2 or 3+ times, 18% were readmitted within 30 days of discharge. Independent variables significantly associated with frequency of hospitalization in the bivariate analyses are presented in Tables 1 to 3*.*

Table 4 presents results for the hierarchical multinomial logistic regression. Concerning needs factors, patients with the highest risk of both moderate or frequent hospitalization rates, as compared with the index group (no hospitalizations), had more chronic physical illnesses, co-occurring MD/SRD, alcohol related disorders, and depressive disorders. Moreover, only patients with moderate hospitalization rates were more likely to be diagnosed with schizophrenia spectrum and other psychotic disorders, and bipolar disorders. Patients with less risk for both frequent and moderate hospitalization rates, as compared with the index group (no hospitalizations), also had more personality disorders, drug related disorders and suicidal behaviors. Only patients with moderate hospitalization rates were less likely to have illness acuity levels 4–5 than patients with no hospitalizations. Regarding predisposing factors, age groups 18–24, 25–44, 45–64, and 65+ years were associated with moderate hospitalizations only, with the 65+ age group most at risk. Patients with frequent hospitalizations only were less likely to visit psychiatric ED integrated with a general ED located in a separate site or a general ED without psychiatric services as compared with the no hospitalizations group. Patients with moderate hospitalizations only were less likely to visit psychiatric ED integrated with a general ED located in a separate site compared with the no hospitalizations group. Enabling factors associated with both frequent and moderate hospitalization rates included consultations with outpatient psychiatrists, prior consultation at ED for any medical condition and number of physicians consulted in specialized care. Particularly high odds ratios were observed for the group making 7+ consultations with outpatient psychiatrists. Only patients with frequent hospitalizations were likely to have more overall interventions (4+) in local community health service centers as compared with patients with no hospitalizations. The final model explained 44% of the variance (Nagelkerke R2). Enabling factors accounted for 28% of the total variance, needs factors 15%, and predisposing factors 1%. The model had also acceptable goodness of fit (Pearson and chi-square statistics (*p* > .05)).

**Table 4:** Estimations of regression coefficients and odds ratios from the multinomial logistic regression model for frequency of patient hospitalizations for any medical condition in 2014–2015. The model reference group is non-hospitalized patients

Variables	Moderate hospitalizations: 1–2 times (2014–15)				Frequent hospitalizations: 3+ times (2014–15)			
	Coefficients	*P*-Value	OR	95% CI	Coefficients	P-Value	OR	95% CI
**Needs factors**								
Mental disorders (MD) (2012–2014)								
Depressive disorders	.31	.00	1.35	1.17–1.57	.34	.01	1.41	1.08–1.84
Schizophrenia spectrum and other psychotic disorders	.19	.00	1.21	1.06–1.38	.23	.05	1.26	1.00–1.58
Bipolar disorders	.28	.00	1.32	1.14–1.52	.23	.09	1.25	.96–1.63
Personality disorders	−.41	.00	.66	.57–.77	−.65	.00	.52	.38–.71
Alcohol related disorders	.33	.00	1.39	1.17–1.64	.41	.01	1.50	1.11–2.04
Drug related disorders	−.29	.00	.75	.63–.89	−.43	.02	.65	.46–.93
Suicidal behaviors in emergency department (ED) visits (2014–2015)	−.24	.00	.79	.72–.87	−.24	.01	.78	.65–.95
Co-occurring MD/substance related disorders (SRD)	.74	.00	2.09	1.85–2.36	1.50	.00	4.45	3.68–5.44
Chronic physical illnesses (2012–2014)	.70	.00	2.02	1.82–2.24	1.64	.00	5.15	4.30–6.18
Illness acuity (triage priority levels) ^a, b^								
Level 3 (urgent)	−.15	.09	.86	.72–1.03	.25	.15	1.29	.91–1.81
Levels 4–5 (less urgent and non-urgent)	−.38	.00	.69	.58–.81	−.29	.15	.75	.51–1.11
**Predisposing factors (2014–15)**								
Age ^b^								
18–24 years	.65	.00	1.91	1.52–2.40	.30	.14	1.35	.91–1.99
25–44 years	.52	.00	1.68	1.35–2.08	.17	.38	1.18	.82–1.17
45–64 years	.60	.00	1.81	1.45–2.26	.05	.79	1.05	.72–1.53
65+ years	.74	.00	2.10	1.64–2.69	.16	.47	1.17	.76–1.81
Deprivation Index:								
Social								
2	−.08	.41	.92	.76–1.12	−.16	.38	.85	.60–1.21
3	−.08	.36	.92	.78–1.09	.03	.86	1.03	.77–1.38
4	−.03	.67	.97	.81–1.14	−.14	.36	.86	.64–1.18
5: Most deprived	.04	.65	1.04	.88–1.22	−.15	.30	.86	.64–1.15
Types of ED ^b^								
Psychiatric ED integrated with a general ED located in a separate site	−.14	.03	.87	.76–.99	−.44	.00	.65	.51–.82
General ED without psychiatric services	.03	.70	1.03	.88–1.20	−.25	.01	.78	.58–1.05
Psychiatric ED merged with general ED	.10	.15	1.10	.97–1.26	−.17	.15	.85	.67–1.06
**Enabling factors** ^**c**^								
Number of consultations for any medical condition with a general practitioner (GP) in medical clinics ^b^								
1–3 consultations	.03	.56	1.03	.93–1.15	−.05	.63	.95	.78–1.16
4+ consultations	−.00	.97	.99	.88–1.13	−.12	.31	.88	.71–1.12
Number of outpatient psychiatrist consultations ^b^								
1–3 consultations	.23	.00	1.26	1.09–1.47	.43	.02	1.54	1.06–2.23
4–6 consultations	.85	.00	2.33	1.92–2.83	1.42	.00	4.13	4.75–6.19
7+ consultations	2.02	.00	7.52	6.45–8.77	3.18	.00	24.00	17.52–33.00
Number of physicians consulted in specialized care ^b^								
1–2 physicians	1.06	.00	2.88	2.50–3.30	.82	.00	2.27	1.53–3.37
3+ physicians	1.20	.00	3.31	2.74–3.99	.59	.00	1.78	1.28–2.53
Prior consultations at ED for any medical condition	.99	.00	2.71	2.42–3.04	.25	.00	11.94	8.16–17.47
Number of interventions in local community service centers ^b^								
1–3 interventions	.02	.72	1.03	.90–1.17	.00	.99	.99	.77–1.30
4+ interventions	−.02	.80	.99	88–1.10	.46	.00	1.58	1.30–1.93

^a^For 0 hospitalizations, illness acuity was calculated considering the average of this data for all ED visits in the index year (2014–15); for 1 hospitalization, in the ED visit leading to this hospitalization; and for 2 and 3+ hospitalizations, the average of these data for the ED visits leading to the 2nd and 3rd hospitalizations

^b^ Reference groups for independent variables with multiple categories were: level 1 and 2 (immediate and very urgent) for illness acuity; 12–17 years for age category; psychiatric ED without medical services within a MH university institute for type of ED; 0 visits for number of consultations for any medical condition with a general practitioner (GP) in medical clinics, number of outpatient psychiatrist consultations, number of physicians consulted in specialized care, and number of interventions in local community health service centers

^c^Measured in the 12 months prior to first ED visit in the index year (2014–2015)

## Discussion

This study identified risk factors for frequency of hospitalization for any medical condition among patients who visited ED at least once in 2014–15 for MH conditions, based on the Andersen Behavioral Model [22]. Results showed that roughly one third of patients had moderate [1, 2] hospitalizations over the 12-month study period, whereas 7% had frequent hospitalizations (3+). Readmission rates in the index year were slightly higher (39%) in this study compared with rates reported in previous research (30–33%) [32, 44]. This difference is easily explained by the inclusion of hospitalizations for both MH and physical illnesses in this study, the latter conditions being more important.

Findings did not confirm the hypothesis that frequent hospitalizations would be more strongly associated with needs factors. Enabling factors accounted for the largest percentage of total variance explained in the study model and were mainly associated with an increased risk for both moderate and frequent hospitalizations even though several needs factors were identified as risk factors.

The finding that prior ED use was a risk factor for increased hospitalization is consistent with the literature [30, 32, 45]. Interestingly, the frequency of outpatient psychiatrist consultations, even as many as 7+, predicted both moderate and frequent hospitalizations, suggesting that these patients had complex conditions (co-occurring disorders and probably important social problems) requiring more extensive and comprehensive care than could be provided by psychiatrist consultations alone. These patients may also have encountered more episodes of exacerbation related to their illnesses and needing inpatient treatment. These hospitalizations may have been planned and may have been shorter, however; but this was unfortunately not measured in this study. Perhaps specialized treatments were also not always adequate to respond to patients with high needs and prevent hospitalization. Research on adequacy of help confirms the difficulty of treating such patients with co-occurring disorders and complex needs. These patients are often viewed as those with “unmet needs” [46, 47]. Even though a third of patients didn’t consult a GP in the 12 months prior to first 2014–15 ED visit, and more than half reported having no family physician, the number of physician consultations in specialized care was found to be associated with increased risk for moderate and frequent hospitalizations. This result was likely associated with the higher rates of chronic physical illnesses in both groups requiring more specialized care.

The single risk factor for frequent hospitalizations only was a high number (4+) of overall interventions in local community health service centers. These centers have reportedly been designated for the treatment of more vulnerable and deprived populations [48]. Some patients may be homeless [49] or affected by other complex conditions, especially psychosocial problems, that may lead to hospitalization due to difficulties involving access to treatment [50, 51]. Moreover, GP working in these centers are known to treat the largest proportion of patients with MD or SRD with complex needs; they thus have greater experience and are used to working in collaboration with MH services [52]. As GP are employed on a salaried basis, as opposed to fee-for-services in private clinics, they are able to dedicate more time to complex patients. Additionally, these centers employ a variety of staff such as social workers, nurses, and nutritionists offering comprehensive care for more vulnerable populations. Training in crisis management and increased multi-modal clinical approaches for staff in these centers may be helpful in reducing hospitalizations as they are likely to be the primary source of care for these patients [52, 53]. Several studies confirm that psychosocial MH interventions and compliance with follow-up directives may reduce the risk of hospitalization [45, 54, 55]; yet such interventions may be insufficient or poorly integrated with other healthcare services to adequately meet the needs of patients with frequent hospitalizations.

Concerning needs factors, the association between diagnoses of depressive disorders with moderate and frequent hospitalizations mirrored the associations with higher rates of hospital readmission reported in previous studies [11, 56, 57]. High rates of co-occurring disorders [16, 58] and poor health outcomes [59, 60] were also frequent characteristics among these patients. Few services are available for Quebec patients with common mental disorders including depressive disorders, with onerous wait times for free counselling services [48]. Alcohol-related disorders [61], withdrawal [62–64], and co-occurring MD/SRD [65, 66] have been shown to elevate the risk of hospital readmission. Higher hospitalization rates may also be explained by higher rates of physical illnesses in patients with co-occurring MD/SRD [25, 67, 68], such as cardiovascular diseases [69], metabolic syndrome [70, 71], obesity [72] and liver diseases [73].

In fact, patients with both frequent and moderate hospitalizations presented more severe and complex conditions than those not hospitalized; but moderate hospitalizations included patients with more serious MD than those who were frequently hospitalized. Surprisingly, serious psychiatric disorders (schizophrenia spectrum and other psychotic disorders or bipolar disorders) predicted moderate hospitalizations only. It may be that patients with serious MD received more intensive ambulatory biopsychosocial care such as assertive community treatment (ACT) and intensive case management, than other patients, as previous Quebec MH reforms have aimed to consolidate services for these patients in particular. ACT and case management have been found to prevent rehospitalization [74]. Unfortunately, such outpatient services were not measured in this study, as the focus was more on physician services and public primary care psychosocial services. However, illness acuity level 4–5 was less likely to be associated with moderate hospitalizations only, indicating that most patients in the “moderate” group were presenting at ED for urgent medical conditions.

Unlike results of other studies [27, 61], this study identified personality disorders, drug related disorders and suicidal behaviors as negative predictors of hospitalization among patients with moderate or frequent hospitalizations compared with no hospitalizations. This situation may be partially due to stigmatizing attitudes among hospital staff toward patients with personality disorders or drug related disorders, considering them less than optimal candidates for hospitalization [75, 76]. Research also indicates that effective treatment for personality disorders involves sound psychiatric management [77] and effective psychological and psychosocial treatments [78, 79], rather than hospitalization. As well, suggested treatments for drug related disorders include opioid agonist treatment integrated with psychosocial treatment, rather than hospitalization [80–82]. Thus, such patients may more likely have been discharged after an ED visit than hospitalized. Differences in study results may also be a function of service availability and the organization of care in various settings [83]. Moreover, personality disorders are often associated with suicidal behaviors [84, 85]. Patients in this study experienced more suicidal ideation than attempts, which may explain their potentially greater frequency of referrals to outpatient services, including crisis centers, over hospitalization. While some studies have found increased risk of hospitalization following instances of suicidal behaviors [23, 55, 65], others found no such association [86, 87].

Concerning predisposing factors, the fact that visiting a psychiatric ED integrated with a general ED in a separate site was associated with lower risk of moderate and frequent hospitalizations may be explained by better coverage and more integrated care in this type of ED as well as increased access to health services, which may have reduced hospital use. The implementation of new assertive community treatment and intensive case management programs targeted to frequently hospitalized patients, under the MH reform, may have directly contributed to this positive outcome. A lower risk of moderate and frequent hospitalizations was also found for patients who had visited the general ED without psychiatric services. The fact that most patients were hospitalized for physical illnesses only may explain their reduced risk of hospitalization.

The association of all age groups, except the 12–17 group, with moderate hospitalizations speaks to the underutilization of services by youth. Low hospitalization rates in the 12–17 age group have also been identified in other studies [24, 88]. While most MD appear at an early age, they are often diagnosed several years after the first symptoms appear. Outpatient services are favored by this group, as young people view hospitalization as very stigmatizing [89]. However, all age groups were represented among those with frequent hospitalizations in this study. Studies have underlined that youth is a predictor for high ED use [90, 91]. Results related to material and social deprivation were not associated with risk of hospitalization in the final model of this study, which coincided with previous studies that have generally reported mixed results [92, 93]. The study results on material deprivation may be explained by the universal Canadian healthcare system, whereas social deprivation did not seem a sufficient risk factor to influence whether such health conditions would lead to hospitalizations.

This study had several limitations that should be mentioned. First, the data were drawn from administrative databanks that were not primarily developed for clinical investigation, but for financial purposes, suggesting that they be interpreted with caution. Second, the data excluded many key variables not available in the Quebec databanks such as ambulatory services delivered by hospitals not provided by physicians, private services such as psychologists, and community-based services which may have contributed to prevent hospitalization. Data on patient medication consumption and on psychopharmacological treatment adherence were also not unavailable for all patients, precluded their use in this study. It is well known that lack of treatment adherence is a key risk factor for hospitalization [94]. As well, the Quebec databanks did not include key variables, such as homelessness [95], race/ethnicity [32, 96] or MD symptom severity, which may have contributed to hospitalization. Third, the interaction between factors not studied may have had an overall impact on study results. Fourth, while all study participants had visited ED in 2014–15 for MH conditions, the sample was not entirely composed of patients diagnosed with MD-SRD. Finally, the results may not be generalizable to other healthcare systems, particularly those without universal coverage as in Quebec/Canada.

## Conclusions

This is the first study to examine risk factors for moderate and frequent hospitalizations compared with no hospitalizations for medical conditions among patients who visited different types of ED for MH conditions in 2014–15, using the Andersen Behavioral Model. Compared with patients who had no hospitalizations or moderate hospitalizations, those with frequent hospitalizations received relatively more interventions overall in local community health service centers, implying that this group consisted of more vulnerable patients with important biopsychosocial problems. Patients with frequent and moderate hospitalizations, compared with no hospitalizations, were also seen more often by outpatient psychiatrists, and had visited more ED previously for medical conditions. Overall, the results suggest that these patients had severe and complex conditions, including co-occurring MD/SRD as well as chronic physical illnesses. Compared with non-hospitalized patients, those with moderate hospitalizations presented more serious MD (schizophrenia spectrum and bipolar disorders), were more than 18 years old, and were less likely to have ED illness acuity levels 4–5. Based on these findings, strategies aimed at reducing frequent hospitalizations need to be provided to these patients, most of whom have co-occurring disorders, with more intensive and diversified biopsychosocial assistance, integrated treatments, and continuous care in response to their multiple needs. For the group with moderate hospitalizations, the focus should be on patients with serious MD, addressing their urgent needs more effectively. Overall, more comprehensive, intensive, and integrated outpatient services for MH-SRD and co-occurring physical illnesses are needed to avoid unnecessary hospitalizations, especially after hospital discharge. Improved access to primary care, both family physicians and local community health service centers, and planning for enhanced outpatient care after discharge are also recommended.

## Data Availability

The datasets analyzed during the current study are available from the corresponding author on reasonable request.

## Abbreviations

BDCU: Banque de données commune des urgences (Quebec emergency databank)
CI: Confidence interval
ED: Emergency department
GP: General practitioner
MD: Mental disorder
MED-ECHO: *Maintenance et exploitation de données pour l’étude de la clientèle hospitalière* (hospitalization/discharge databank)
MH: Mental health
SD: Standard deviation
SRD: Substance related disorder
SUD: Substance use disorder
RAMQ: *Régie d’Assurance Maladie du Québec* (Quebec healthcare insurance)
